# The Interrelationship between Cadmium, Smoking, and Migraine in the ELSA-Brasil Study

**DOI:** 10.1007/s12011-026-05308-x

**Published:** 2026-09-01

**Authors:** Arão Belitardo de Oliveira, Fernando Barbosa, Andrea Carmine Belin, Joseph O. Lloyd, Itamar S. Santos, Mario Fernando Prieto Peres, Paulo A. Lotufo, Isabela M. Benseñor, Alessandra C. Goulart

**Affiliations:** 1 Center for Clinical and Epidemiological Research, Hospital Universitario, Universidade de Sao Paulo, São Paulo, Brazil; 2 School of Public Health, Department of Epidemiology, University of Sao Paulo, São Paulo, Brazil; 3 Laboratório de Toxicologia Analítica e de Sistemas (ASTOx). Departamento de Análises Clínicas, Toxicológicas e Bromatológicas, Faculdade de Ciências Farmacêuticas de Ribeirão Preto, Universidade de São Paulo, Ribeirão Preto, Brazil; 4 Centre for Cluster Headache, Department of Neuroscience, Karolinska Institutet, Stockholm, Sweden; 5 Department of Internal Medicine, School of Medicine, Universidade de Sao Paulo, São Paulo, Brazil; 6 Instituto do Cérebro, Hospital Israelita Albert Einstein, São Paulo, Brazil; 7 Instituto de Psiquiatria, Hospital das Clínicas, Universidade de São Paulo, São Paulo, Brazil

**Keywords:** Migraine, Smoking, Heavy metal, Cadmium

## Abstract

This study explored the relationship between serum cadmium (Cd), smoking exposure, and migraine in the ELSA-Brasil cohort (2008–2010). The analysis included 2,750 participants whose serum Cd levels were measured using inductively coupled plasma mass spectrometry. Smoking exposure was assessed using self-report data of active smoking and second-hand smoke. Migraine, including definite and probable migraine, was diagnosed according to the International Classification of Headache Disorders, 3rd edition (ICHD-3). Logistic regression was used to estimate the odds of migraine across serum Cd quintiles, using the third quintile as the reference category, while linear regression examined the relationship between smoking exposure and Cd levels. Polynomial contrasts tested linear trends. Participants had a mean (SD) age of 53.2 (9.0) years and 53.1% were women. Migraine prevalence was 29.1% (including probable migraine). Individuals with migraine had slightly higher median serum Cd levels than controls [0.050 µg/L (IQR 0.035–0.077) vs. 0.048 µg/L (0.035–0.066); *p* = 0.021], although smoking exposure scores did not differ between groups. Smoking exposure showed a strong positive association with serum Cd concentrations (p-trend < 0.001). Participants in the highest Cd quintile had greater odds of migraine [aOR 1.51 (95% CI 1.09–2.08), *p* = 0.011] after adjustment for smoking exposure and sociodemographic and clinical confounders. Sex-stratified analysis yielded even stronger associations among males [aOR 1.84 (95% CI 1.07–3.16), *p* = 0.027]. However, in the sensitivity analysis including definite migraine cases only, this association was no longer significant [aOR: 1.14 (0.71, 1.83), *p* = 0.579]. Main findings suggest that Cd exposure from smoking may contribute to migraine occurrence, particularly in males. However, other sources of Cd that could influence migraine should not be disregarded, and future investigation in this field is warranted.

## Introduction

Migraine has been steadily ranked as a leading cause of disability worldwide over the past 30 years [1]. According to the Global Burden of Disease Study 2023 [1], around 14% of the global population suffers from migraine, whereas in terms of years lived with disability, migraine is second to stroke as the most disabling neurological disease in the world [2].

Migraine is influenced by complex and still poorly understood interactions between endogenous, lifestyle, and environmental factors [3, 4]. Emerging evidence suggests that exposure to some metals is associated with migraine or headache attack frequency [5–7]. Likewise, smoking is associated with a higher risk of migraine, albeit less is known regarding passive smoking [8]. It has long been acknowledged that tobacco smoking is the main source of cadmium (Cd) accumulation in the body [9, 10]. An observational study with a small sample size (*n* = 50) reported higher serum Cd levels in migraine patients compared to non-migraine counterparts [6]. However, no study has yet explored whether smoking-related Cd would be associated with migraine, or rather whether Cd would be independently associated with migraine regardless of smoking status or passive smoking. Studies indicate a sex-specific pattern in Cd bioaccumulation, with women exhibiting higher body burdens than men at equivalent exposures [11, 12]. This disparity is potentially mediated by physiological factors, including enhanced divalent metal transporter 1 expression and reduced iron reserves in women [10–12].

Furthermore, the pharmacological management of migraine introduces complex and opposing biases that must be rigorously addressed when analyzing the association between migraine prevalence and serum Cd. Preventative medications (e.g., beta-blockers, antiepileptics, antidepressants) are predominantly prescribed for other comorbidities such as hypertension, diabetes, and depression, thereby acting as confounders of migraine prevalence estimates [13]. Abortive medications such as non-steroidal anti-inflammatory drugs (NSAIDs) (e.g., paracetamol, acetylsalicylic acid, ibuprofen.) are commonly used by migraine patients and may contain trace Cd contaminants [14], potentially placing them on the causal pathway between migraine and Cd exposure. No study so far has considered these confounders in the migraine-Cd link.

Therefore, we aimed to address this research gap by exploring the associations between Cd, migraine, and smoking exposure, considering several potential behavioral and environmental confounders. Owing to sex differences in migraine prevalence [3] and Cd metabolization [10], we wondered whether there would be sex-specific associations.

We hypothesized that smoking would be associated with higher serum Cd levels, and these higher serum Cd levels would be associated with higher odds of migraine in the Brazilian Longitudinal Study of Adult Health (ELSA-Brasil). Furthermore, we predict that these associations would be stronger among females.

## Methods

### Study Design and Participants

This is a cross-sectional study with secondary, post hoc analysis of a subset of participants from the ELSA-Brasil study. In brief, the ELSA-Brasil study is a large ongoing multicenter cohort, designated to investigate several lifestyle and risk factors associated with major non-communicable diseases in a sample of 15,105 civil servants (35 to 74 years of age) from six metropolitan areas of Brazil: Belo Horizonte, Porto Alegre, Rio de Janeiro, Salvador, São Paulo, and Vitória [15]. Although the primary aim of ELSA-Brasil is to investigate the incidence of diabetes, cardiovascular diseases, and associated risk factors, the study has also examined other comorbidities, including headache disorders, lifestyle factors, and an array of laboratory tests, including serum metals/metalloids [15, 16].

From 5,061 individuals from the ELSA-Brasil (São Paulo’s Investigation Center) with migraine data, we considered 2,750 with full data on serum Cd, headache disorders, and tobacco smoking or passive smoking at baseline (2008–2010). We analyzed data from 2,750 participants.

The São Paulo University Hospital’s Research Ethics Committee (CEP-HU/USP:#659/06) and the National Research Ethics Committee (CAAE: #08109612.7.1001.0076) approved the study, and informed consent was provided previously to the beginning of the study. This study complies with the Strengthening the Reporting of Observational studies in Epidemiology’s guidelines.

### Primary Outcome and Exposures

Although this is a cross-sectional analysis, serum Cd and its association with smoking exposure were set as the exposure/predictor, and the primary outcome was migraine diagnosis.

### Migraine Diagnosis - Outcome

At the baseline of the ELSA-Brasil study, migraine diagnosis was determined upon a structured headache questionnaire based on the International Classification of Headache Disorders (ICHD), 2nd Edition [17] previously validated in Brazil [18]. The classification of “probable migraine” was applied to individuals who fulfilled all but one of the required diagnostic criteria. Participants experiencing no headache or other primary non-migraine headaches in the past 12 months were classified as “No Migraine”.

Telephone interviews were conducted by an expert medical doctor from the ELSA-Brasil study, who established all diagnoses according to the 3rd edition of the ICHD [19].

### Serum Cd Levels - Exposure

Prior to analysis, plasma samples were diluted 1:20 with a solution of 0.5% v/v HNO₃ and 0.005% v/v Triton X-100. Serum Cd levels were quantified using inductively coupled plasma mass spectrometry (ICP-MS; NexION^®^ 2000, PerkinElmer, Shelton, CT, USA) equipped with a Quadrupole Ion Deflector, following the method of Batista et al. (2009) [20]. The accuracy and precision of the data were verified by measuring reference serum samples from the *Institut National de Santé Publique du Québec* (Quebec, Canada). Results agreed with target values (t-test, 95%). Measurements below the limit of detection (LOD) were imputed as LOD/√2, and serum Cd levels are expressed in micrograms per litre (µg/L).

### Smoking - Exposure

The smoking exposure was determined by self-reported information of participants’ smoking status and exposure to second-hand smoke. We categorized the smoking exposure based on the Life’s Essential 8 (LE8) scoring system, proposed by the American Heart Association (AHA) [21]. A score of 100 points is assigned for being a never-smoker, 75 points for being a former smoker who quit six or more years ago, 50 points for having quit between two and six years ago, and 25 points for having quit for less than two years [21]. A base score of 0 points is assigned to a current smoker. This base score is then adjusted for exposure to secondhand smoke: 20 points are deducted if the individual lives with an active indoor smoker, unless the score is already 0 [21]. As such, the lower the score, the higher the exposure. For this study, the smoking exposure score was categorized into 3 levels of exposure: high exposure (Score < 50), moderate (Score 50–79), and low exposure (Score ≥ 80), following AHA LE8 score categories [21].

### Other Covariates

Covariates included sociodemographic factors, lifestyle, and use of medication with acute and prophylactic actions for migraine. Sociodemographic variables encompassed sex assigned at birth (female or male), age (years), household income (continuous values *per capita*, expressed in US$), educational level (elementary, high school, or college), race determined by self-identified skin color (White, Black, Brown or Pardo, Others – Indigenous, Asian), and marital status (married, separated, single, widow/widower, or other).

Alcohol consumption (no, former, current) and continuous body mass index (BMI) were selected as covariates for lifestyle and health factors.

Alcohol consumption was assessed using the validated, semiquantitative ELSA-Brasil Food Frequency Questionnaire [22]. Beverages were listed separately to determine type consumed, and intake was calculated in mL/day, kcal/day, and grams of ethanol per day (g/day) for each beverage type. Ethanol content was based on average alcohol levels of common Brazilian brands: beer 5%, wine 12%, and distilled spirits 39% [23]. Using ethanol density (0.8 g/cm³), pure alcohol intake from all beverage types per participant was calculated as weekly volume (mL) × alcohol content × 0.8 and expressed as g/week [23].

To address the effects of preventive and acute medication on migraine prevalence and Cd levels, we included available data regarding common prophylactic and abortive medications for migraine in the adjustments in the ELSA-Brasil. The self-reported use of any medication with prophylactic and abortive effects on migraine (yes/no) included preventive medications with evidence level A and B, according to the American Academy of Neurology’s guidelines [24] and common acute medications used in Brazil and/or recommended by the American Headache Society’s Consensus [13] The prophylactic medications included for each evidence level were: Level A: antiseizure drugs (divalproex sodium, sodium valproate, topiramate), beta-blockers (propranolol, metoprolol, timolol); Level B: antidepressants (amitriptyline, venlafaxine) and beta-blockers (atenolol, nadolol). The current use of any abortive medications (yes/no) for migraine included NSAIDs (e.g., paracetamol, acetylsalicylic acid, ibuprofen, dipyrone), triptans, ergotamines, and opioids [13].

## Statistics

Descriptive statistics for sociodemographic, clinical, and laboratory data are presented as absolute values and percentages (%), mean with standard deviations (± SD), or median with interquartile range (IQR). Inspecting the histograms and Kernel density plots verified adherence to the assumption of a normal distribution. Comparison between migraine diagnoses (no migraine vs. migraine) and sex (males vs. females) groups was conducted using the Chi-square test, t-test for independent samples, and the Wilcoxon rank-sum test, depending on whether variables were categorical, continuous, or non-normally distributed, respectively.

For the primary outcome in this study, binary logistic regression was fitted to estimate the odds ratio (OR) with 95% confidence interval (CI) of migraine diagnoses (outcome, “no” set as reference) based on quintiles (Q) of serum Cd levels at baseline (predictor, Q3rd set as reference). Adjusted models controlled for the effects of the sociodemographic variables age (continuous), sex (“female” as reference, and “male”), race self-reported skin color (“White” as reference, “Brown”, “Black”, and “Others”), household income (continuous values per capita, expressed in US$), schooling (“College” as reference, “High School”, and “Elementary”), marital status (“married” as reference, “single”, “separated/divorced”, and “widow[er]”), BMI (continuous), smoking scores (continuous) and alcohol consumption (“never” set as reference, “former”, and “current”), and use of any class of migraine prophylactic and acute medications (“no” set as reference, and “yes” if participants currently used at least one medication class).

To test whether sex was an effect modifier, sex interactions were computed across models, and male was set as the reference. Additionally, we implemented sex- and smoking exposure-stratified analysis. In the analysis stratified by smoking exposure, we used the 3 categories of exposure, namely, high exposure (< 50), moderate (50–79), and low exposure (≥ 80).

The relationships between tobacco smoking exposure score and serum Cd quintiles, as well as between migraine and serum Cd quintiles, were formally tested for linearity. After fitting the linear and binary regression models, we used the polynomial contrasts feature of Stata’s contrast command to decompose the trend across Cd quintiles into its orthogonal components. The statistically significant associations were plotted using the *marginsplot* command to visually illustrate the predicted values of dependent variables based on independent variables.

To test whether the model significantly improved fit over the null model, the Hosmer-Lemeshow goodness-of-fit test was conducted. The model demonstrated a significantly better fit to the data than the null model (*p* = 0.004), and the Hosmer-Lemeshow test indicated that the model adequately fit the data (*p* = 0.216). We assessed multicollinearity using the variance inflation factor (VIF); the VIF value was 1.10, well below the common threshold of 5 or 10, confirming that multicollinearity was not a concern.

A sensitivity analysis of the logistic regression model of the primary outcome was conducted by excluding probable migraine cases (*n* = 492).

Data were analyzed in Stata version 17.0 (StataCorp LLC, College Station, TX, USA). A two-tailed p-value < 0.05 was considered statistically significant for all tests.

## Results

**Fig. 1:**
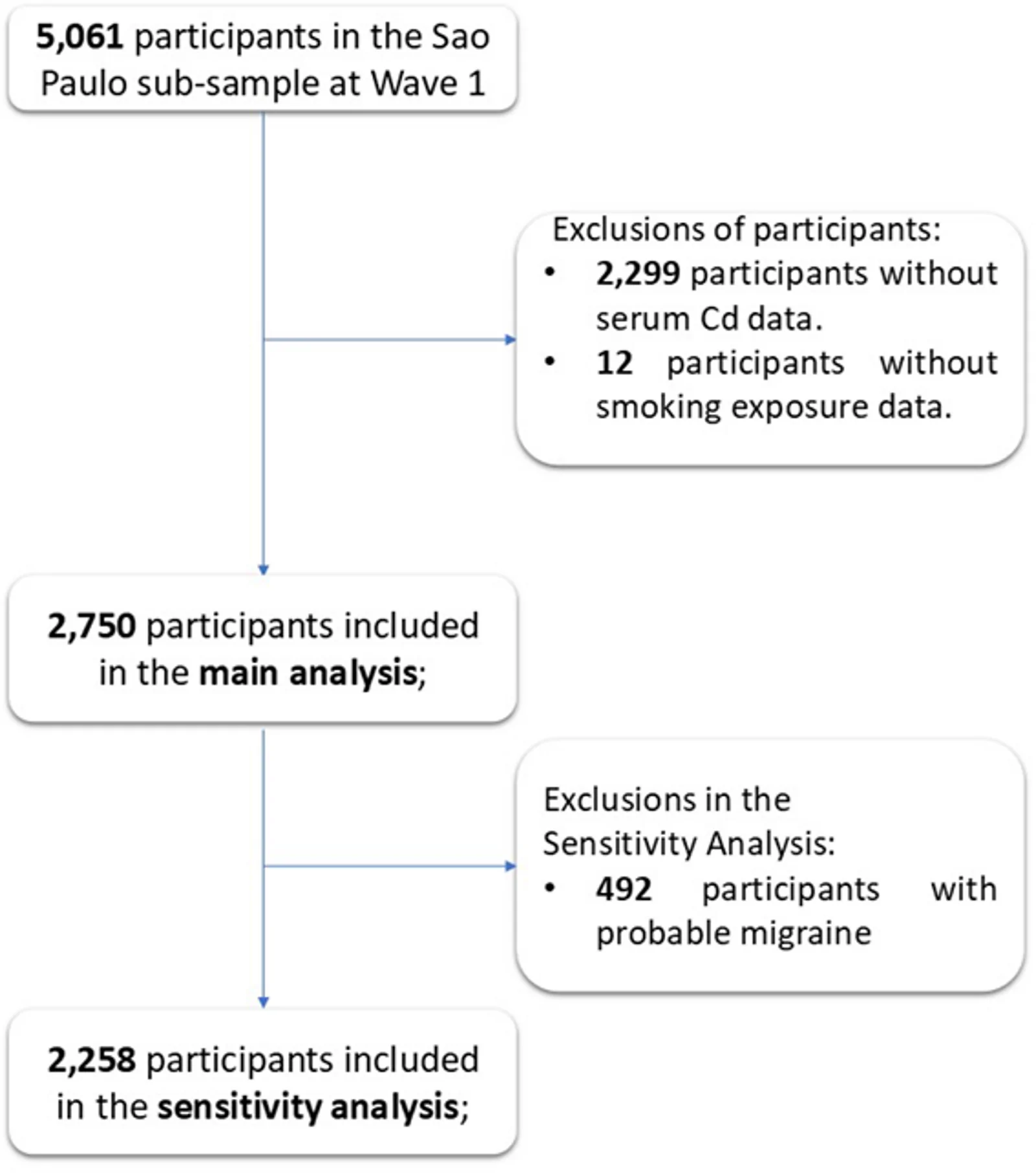
Participants’ flow in the study

In this study, 2,750 participants provided full data on the selected variables and were analyzed (Fig. 1). The sample’s mean (SD) age was 53.2 (9.0) years, and 53.1% (1,462/2,750) were female. The prevalence of any migraine (migraine and probable migraine) was 29.2% (803/2,750), while 11.3% (311/2,750) presented definite migraine.

Sociodemographic data, laboratory results, clinical characteristics, and lifestyle factors at baseline are reported in Table 1. Participants with migraine were predominantly female, non-white, younger, belonged to lower socioeconomic strata, had mostly middle levels of educational attainment, and consumed less alcohol, compared to the no migraine group (Table 1).

**Table 1:** Participants’ characteristics in the ELSA-Brasil study at baseline (*n* = 2,750)

	No migraine (*n* = 1,947)	Any migraine (*n* = 803)	*p*-value
Age, years, Mean (SD)	53.3 (9.2)	49.7 (8.0)	< 0.001
BMI, kg/m^2^, Mean (SD)	27.2 (4.8)	27.3 (5.0)	0.694
Sex, n (%)			
Female	858 (44.0)	604 (75.2)	< 0.001
Ethnicity - self-reported, n (%)			
White	1,151 (59.1)	216 (54.7)	0.025
Brown	391 (20.0)	93 (24.1)	
Black	260 (13.3)	123 (15.3)	
Other	114 (5.8)	38 (4.7)	
Education, n (%)			
Elementary	368 (18.9)	118 (14.6)	< 0.001
High School	683 (35.0)	370 (46.0)	
College	896 (46.0)	315 (39.2)	
Monthly Income (*per capita*)^a^, Median US$ (IQR)	622.45 (345.75, 1,141.10)	539.45 (311.12, 933.62)	< 0.001
Marital Status, n (%)			
Married	1,325 (68.0)	263 (65.2)	0.450
Separated	280 (14.3)	118 (14.6)	
Single	203 (10.4)	88 (10.9)	
Widow(er)	91 (4.6)	47 (5.8)	
Other	48 (2.4)	26 (3.2)	
Use of Migraine Medication			
Acute, n (%)	174 (8.9)	407 (50.6)	< 0.001
Preventive, n (%)	222 (11.4)	83 (10.3)	0.397
Alcohol Consumption, n (%)			
Never	227 (11.6)	122 (15.2)	
Former	374 (19.2)	218 (27.2)	< 0.001
Current	1,346 (69.2)	463 (57.6)	
Median (IQR) intake (g/week)	35.0 (0, 105.0)	18.7 (0, 64.8)	< 0.001
Smoking Exposure Levels, n(%)			
Low (≥ 80)	1,019 (52.3)	449 (55.9)	
Moderate (50–79)	554 (28.5)	201 (25.0)	0.150
High (< 50)	374 (19.2)	153 (19.1)	

^a^Based on a 2:1 exchange rate between US dollar and Real in the study period. Missing values accounted for less than 5% for race, income, and acute and preventive medications, therefore, the total percentage is not 100% in these variables’ columns

The migraine group showed higher median (IQR) values of Cd [0.050 µg/L (0.035, 0.077) vs. 0.048 µg/L (0.035, 0.066), *p* = 0.021], while there was no between-group difference for smoking exposure score [80 (55, 100) vs. 80 (55, 100), *p* = 0.527]. Likewise, smoking exposure levels did not differ significantly between the migraine and no migraine groups (*X*^2^(2) = 3.79, *p* = 0.150) (Table 1). Females showed higher median (IQR) values of Cd [ 0.049 µg/L (0.035, 0.071) vs. 0.047 µg/L (0.034, 0.067), *p* = 0.009] and smoking exposure score [100 (75, 100) vs. 75 (55, 100), *p* < 0.001] than males.

**Fig. 2:**
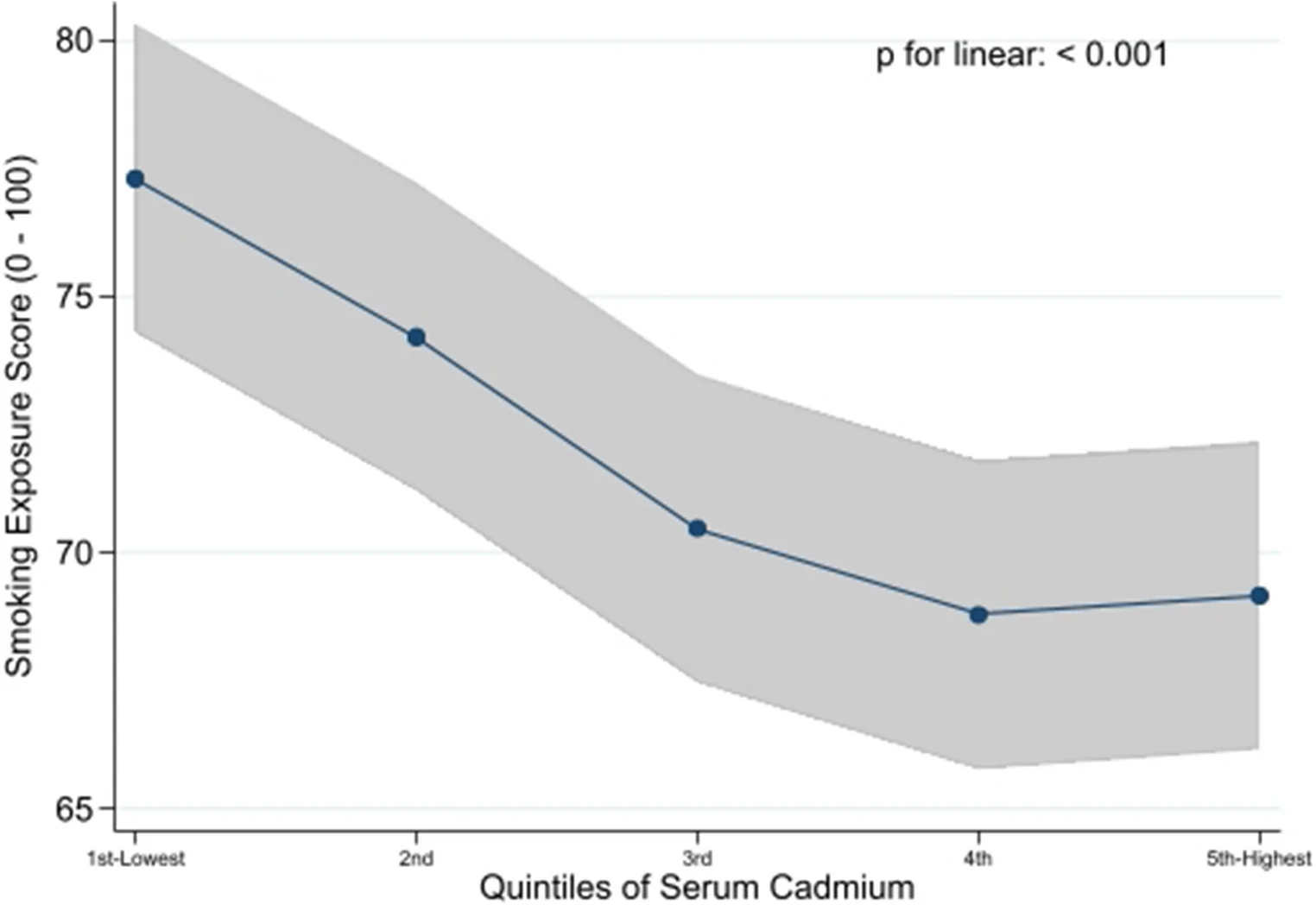
Associations between smoking exposure score* and serum Cd levels in the ELSA-Brasil study (*n* = 2,750). *: The higher the score, the healthier; The lower the score, the higher the smoking exposure

In the whole sample, the orthogonal polynomial contrast for a linear trend was statistically significant (p-trend < 0.001), indicating that as smoking exposure scores decreased (meaning increased exposure), serum Cd levels increased linearly (Fig. 2).

**Fig. 3:**
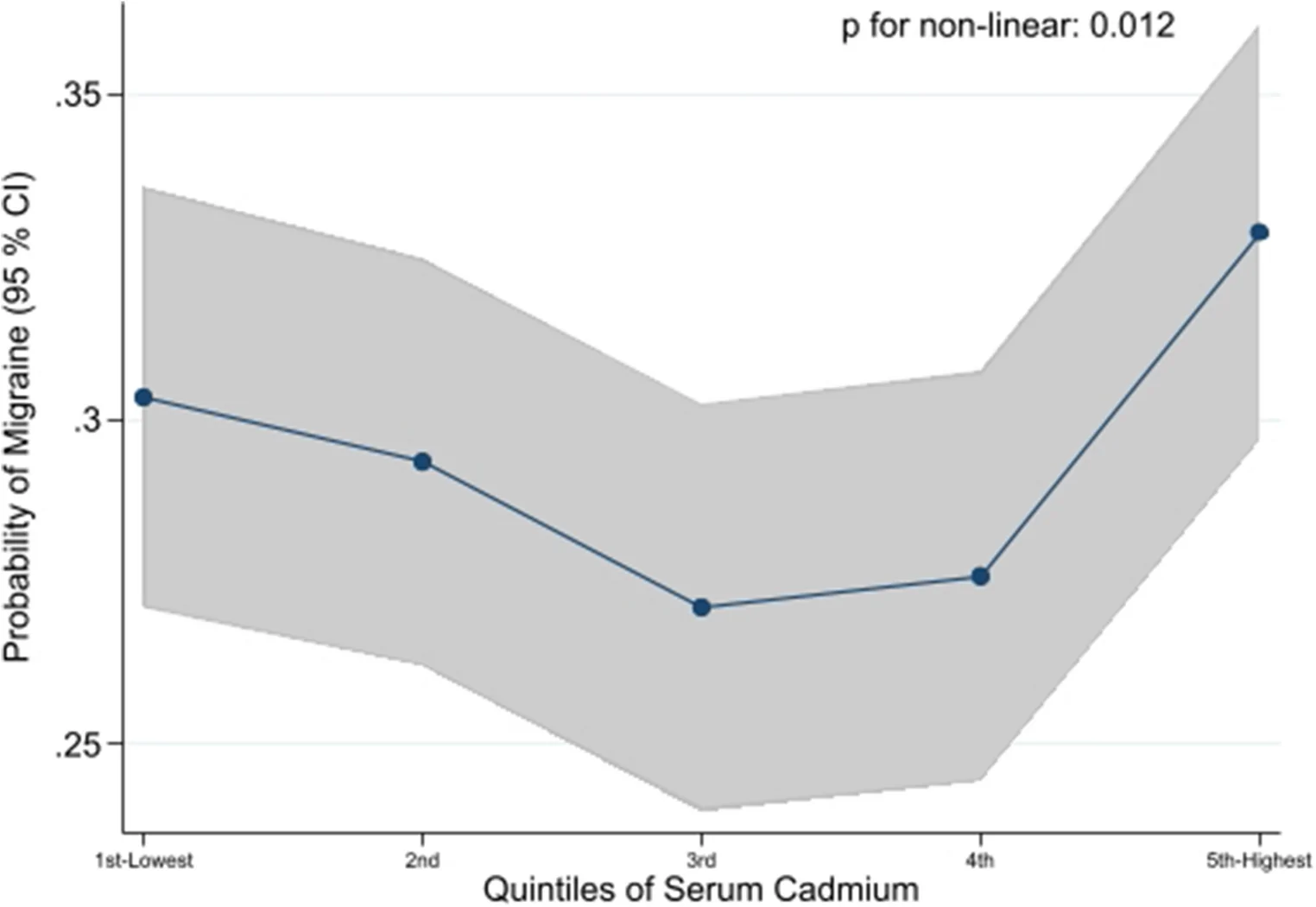
Associations between migraine diagnosis and serum Cd levels in the ELSA-Brasil study (*n* = 2,750)

Based on the primary outcome binary regression models, the highest quintile (Q5) of serum Cd levels was associated with increased odds of migraine [OR: 1.79 (1.38, 2.33), *p* < 0.001] compared with the 3rd quintile (Q3) (Table 2). This pattern of association remained significant even after the adjustments for confounders, including smoking exposure [aOR: 1.51 (1.09, 2.08), *p* = 0.011] (Table 2). The quadratic component was statistically significant (*p* = 0.012), revealing that the relationship between Cd quintiles and migraine was curved, with a “J-shaped” feature (Fig. 3).

**Table 2:** Odds ratio (OR) with 95% confidence interval (CI) for any migraine by quintiles (Qs) of serum Cd among 2,750 participants from the ELSA-Brasil study at baseline

	Serum Cd quintiles	OR	(95% CI)	*p*-value
Crude Model	Q1 (Lowest)	1.39	(0.81, 2.40)	0.223
	Q2	1.23	(0.70, 2.14)	0.462
	Q3	Ref. (1.0)	Ref. (1.0)	Ref. (1.0)
	Q4	0.93	(0.51, 1.69)	0.833
	Q5 (Highest)	**1.79**	**(1.38**,** 2.33)**	**< 0.001**
Adjusted Model	Q1 (Lowest)	1.24	(0.89, 1.73)	0.190
	Q2	1.17	(0.84, 1.63)	0.326
	Q3	Ref. (1.0)	Ref. (1.0)	Ref. (1.0)
	Q4	1.03	(0.73, 1.44)	0.844
	Q5 (Highest)	**1.51**	**(1.09**,** 2.08)**	**0.011**

Reference group: No Migraine (*n* = 1,947); The adjusted model controlled for sex, age, BMI, race, income *per capita*, schooling, marital status, smoking exposure score, alcohol consumption, and use of migraine prophylactic and acute medications

There was no significant sex interaction effect in the adjusted models (p-interaction = 0.725).

However, the sex-stratified analysis revealed that the pattern of association regarding the highest quintile of Cd remained significant for males [OR: 1.84 (1.07, 3.16), *p* = 0.027] but not females [OR: 1.39 (0.92, 2.09), *p* = 0.110] in the adjusted models (Table 3).

**Table 3:** Sex-stratified analysis of the association between serum Cd and any migraine in the ELSA-Brasil study (*n* = 2,750) at baseline

	Serum Cd quintiles	OR	(95% CI)	*p*-value
	Females (*n* = 1,462)			
Crude Model	Q1 (Lowest)	1.19	(0.84, 1.69)	0.307
	Q2	1.19	(0.85, 1.67)	0.300
	Q3	Ref. (1.0)	Ref. (1.0)	Ref. (1.0)
	Q4	1.19	(0.85, 1.67)	0.300
	Q5 (Highest)	**1.63**	**(1.17**,** 2.27)**	**0.003**
Adjusted Model	Q1 (Lowest)	1.18	(0.77, 1.82)	0.428
	Q2	1.14	(0.75, 1.74)	0.515
	Q3	Ref. (1.0)	Ref. (1.0)	Ref. (1.0)
	Q4	1.06	(0.69, 1.61)	0.786
	Q5 (Highest)	1.39	(0.92, 2.09)	0.110
	Males (*n* = 1,288)			
Crude Model	Q1 (Lowest)	1.39	(0.86, 2.24)	0.172
	Q2	1.30	(0.79, 2.13)	0.293
	Q3	Ref. (1.0)	Ref. (1.0)	Ref. (1.0)
	Q4	0.93	(0.55, 1.58)	0.810
	Q5 (Highest)	**1.85**	**(1.15**,** 3.00)**	**0.011**
Adjusted Model	Q1 (Lowest)	1.37	(0.79, 2.36)	0.253
	Q2	1.22	(0.70, 2.13)	0.475
	Q3	Ref. (1.0)	Ref. (1.0)	Ref. (1.0)
	Q4	0.92	(0.51, 1.66)	0.787
	Q5 (Highest)	**1.84**	**(1.07**,** 3.16)**	**0.027**

Reference group: No Migraine (Females, *n* = 858; Males, *n* = 1,089); The adjusted model controlled for smoking exposure score, age, BMI, race, income *per capita*, schooling, marital status, alcohol consumption, and use of migraine prophylactic and acute medications

In the analysis stratified by smoking exposure levels, the unadjusted models showed progressively stronger associations with increasing exposure (Table 4). In the adjusted models, these associations were not significant (Table 4).

**Table 4:** Smoking exposure-stratified analysis of the association between serum Cd and any migraine in the ELSA-Brasil study (*n* = 2,750) at baseline

	Serum Cd quintiles	OR	(95% CI)	*p*-value
	Low Smoking Exposure – Score ≥ 80 (*n* = 1,468)			
Crude Model	Q1 (Lowest)	1.19	(0.84, 1.70)	0.319
	Q2	1.16	(0.81, 1.67)	0.412
	Q3	Ref. (1.0)	Ref. (1.0)	Ref. (1.0)
	Q4	1.31	(0.91, 1.90)	0.141
	Q5 (Highest)	**1.69**	**(1.19**,** 2.41)**	**0.003**
Adjusted Model	Q1 (Lowest)	1.24	(0.81, 1.91)	0.314
	Q2	0.97	(0.62, 1.51)	0.914
	Q3	Ref. (1.0)	Ref. (1.0)	Ref. (1.0)
	Q4	1.07	(0.68, 1.70)	0.742
	Q5 (Highest)	1.38	(0.90, 2.14)	0.137
	Moderate Smoking Exposure – Score 50–79 (*n* = 755)			
Crude Model	Q1 (Lowest)	1.25	(0.73, 2.14)	0.399
	Q2	1.28	(0.76, 2.17)	0.344
	Q3	Ref. (1.0)	Ref. (1.0)	Ref. (1.0)
	Q4	0.86	(0.49, 1.49)	0.600
	Q5 (Highest)	**1.89**	**(1.12**,** 3.20)**	**0.017**
Adjusted Model	Q1 (Lowest)	1.27	(0.65, 2.50)	0.477
	Q2	1.31	(0.68, 2.55)	0.410
	Q3	Ref. (1.0)	Ref. (1.0)	Ref. (1.0)
	Q4	0.72	(0.36, 1.47)	0.379
	Q5 (Highest)	1.36	(0.70, 2.66)	0.353
	High Smoking Exposure – Score < 50 (*n* = 527)			
Crude Model	Q1 (Lowest)	1.09	(0.55, 2.16)	0.803
	Q2	1.55	(0.84, 2.88)	0.158
	Q3	Ref. (1.0)	Ref. (1.0)	Ref. (1.0)
	Q4	1.23	(0.87, 3.85)	0. 105
	Q5 (Highest)	**1.96**	**(1.15**,** 3.00)**	**0.019**
Adjusted Model	Q1 (Lowest)	1.09	(0.44, 2.66)	0.845
	Q2	1.56	(0.71, 3.42)	0.265
	Q3	Ref. (1.0)	Ref. (1.0)	Ref. (1.0)
	Q4	1.43	(0.67, 3.04)	0.342
	Q5 (Highest)	1.84	(0.87, 3.85)	0.105

Reference group: No Migraine (Low, *n* = 1,019; Moderate, *n* = 544; High, *n* = 374); The adjusted model controlled for sex, age, BMI, race, income *per capita*, schooling, marital status, alcohol consumption, and use of migraine prophylactic and acute medications

In the sensitivity analysis that included definite migraine cases only (*n* = 311), the same J-shaped pattern of association persisted. There was an even stronger association between the highest quintile of Cd levels and migraine [OR: 1.86 (1.28, 2.69), *p* = 0.001] compared to the 3rd quartile (Table 5). However, when adjusted for smoking exposure score and other sociodemographic, clinical, and lifestyle variables, the significance was no longer significant [aOR: 1.14 (0.71, 1.83), *p* = 0.579] (Table 5). As in the main analysis, there was no sex-by-interaction effect in the adjusted models (p-interaction = 0.852). Sex- and smoking-stratified sensitivity analyses were precluded by the low count of male participants with migraine (*n* = 7), and absent data in the lowest Cd quintile.

**Table 5:** Sensitivity analysis excluding probable migraine cases for the association between serum Cd and definite migraine in the ELSA-Brasil study (*n* = 2,258) at baseline

	Serum Cd quintiles	OR	(95% CI)	, *p*-value
Crude Model	Q1 (Lowest)	1.17	(0.79, 1.730)	0.432
	Q2	1.04	(0.69, 1.55)	0.843
	Q3	Ref. (1.0)	Ref. (1.0)	Ref. (1.0)
	Q4	1.15	(0.77, 1.70)	0.478
	**Q5 (Highest)**	**1.86**	**(1.28**,** 2.69)**	**0.001**
Adjusted Model	Q1 (Lowest)	1.09	(0.67, 1.78)	0.712
	Q2	0.71	(0.43, 1.19)	0.202
	Q3	Ref. (1.0)	Ref. (1.0)	Ref. (1.0)
	Q4	0.97	(0.59, 1.60)	0.936
	**Q5 (Highest)**	1.14	(0.71, 1.83)	0.579

Reference group: No Migraine (*n* = 1,947); The adjusted model controlled for sex, age, BMI, race, income *per capita*, schooling, marital status, smoking exposure score, alcohol consumption, and use of migraine prophylactic and acute medications

## Discussion

In this large, cross-sectional study, we aimed at exploring the relationship between serum Cd, smoking, and migraine. While we confirmed a strong, inverse linear relationship between smoking exposure scores (increased exposure) and serum Cd, we found that the association between Cd and migraine was influenced by smoking exposure, indicating for the first time the participation of this metal in the migraine-smoking link.

Although our main findings were not confirmed by sensitivity analysis, excluding probable migraine cases, and the effect of Cd can be confounded or fully mediated by smoking exposure in definite migraine cases, the overall and sex-stratified analyses including probable migraine cases yielded significant associations regardless of smoking exposure, particularly in men. This is possibly due to the considerably larger sample, which increased the power to capture the effect of other Cd exposure sources. It is worth mentioning at this stage that 80.7% of probable migraine cases in this sample missed only one of the migraine criteria for definite migraine (all reported < 4 h), likely due to early acute medication use, which may explain why the main analysis retained a significant Cd-migraine association beyond smoking.

This still has clinical and epidemiological relevance in the Cd-migraine link, since population-based studies in Brazil indicate that probable migraine also carries along considerable disability [25, 26], with up to 50% of thse cases being eligible to take migraine preventative medication and around a third of cases reaching the highest disability scores [25]. This underscores the need for further studies to investigate other Cd sources in this relationship.

Yet, our findings concur with a previous small study that reported higher Cd levels in patients with migraine [6], whereas we added further evidence for the participation of smoking-related Cd exposure in migraine pathophysiology.

Taken together, our findings support the role of Cd as a potential contributor to migraine disorders, with smoking emerging as a clear exposure route. They also highlight the need for future investigations to systematically assess sex-specific vulnerabilities, which may arise from differential dietary habits (e.g., consumption of leafy greens, shellfish, organ meats, sunflower seeds, and peanuts), occupational settings (e.g., mining, smelting, battery and plastics manufacturing, and combustion processes), and ambient air pollution in large urban centers [10]. Indeed, the ELSA-Brasil cohort, composed predominantly of residents from major metropolitan areas, has previously shown associations between migraine and both occupational and dietary factors [7, 27, 28].

To add complexity in the migraine-smoking-Cd link, Cd exposure has been associated with a range of epigenetic alterations, including changes in DNA methylation patterns, histone modifications, and dysregulation of non-coding RNAs [29]. In addition, cigarette smoking itself is known to induce epigenetic modifications that may persist for decades after smoking cessation, with detectable signatures reported up to 30 years later [30]. Collectively, these epigenetic perturbations may influence gene expression profiles and thereby contribute to disease development.

Metallothioneins (MT) are a family of metal-sequestering enzymes that maintain homeostasis of essential physiological metals, such as zinc and copper. As well as this, MT captures heavy metals, such as Cd, lead, mercury and arsenic, to prevent toxicity and protect against oxidative stress [31]Therefore, their increased expression can be used as an indicator of heavy metal exposure and could be further studied in this patient group. In mammals, MT exists in four isoforms; MT1 and MT2 are expressed throughout most tissues within the body, MT3 is expressed within the central nervous system, and MT4 is expressed in stratified epithelium [32]. Given the importance of the central nervous system in migraine, expression of MT3 could be an interesting focus in future studies, compared with MT1 and MT2 and markers of total body heavy metal exposure.

This study has several strengths, including a large, well-characterized sample from the multicenter ELSA-Brasil cohort, which enhances generalizability. Serum cadmium was measured using high-precision ICP-MS, and migraine diagnoses were rigorously established through validated ICHD-3-based criteria. We adjusted for an extensive set of confounders, which have been largely overlooked in prior research. The use of the LE8 score enabled us to capture both active and passive smoking, while the formal assessment of non-linear trends further underscores the methodological robustness of our approach.

Our study has several limitations. The cross-sectional design precludes any determination of causality; we cannot establish whether higher Cd levels predispose individuals to migraine or whether people with migraine have behaviors/activities or physiological differences that lead to greater Cd accumulation. The low count of male participants with migraine (*n* = 31) in the main analysis imposes problems with statistically underpowered and unreliable estimates. Furthermore, while we adjusted for numerous confounders, residual confounding from unmeasured factors, such as dietary habits or occupational exposures, cannot be entirely ruled out. The use of a single Cd measurement may not fully reflect long-term body burden, as Cd has a very long half-life, though it is a widely accepted biomarker of recent exposure [10]. Finally, the formal interaction tests revealed no statistically significant sex differences, indicating that the stratified results could be due to random variation or spurious associations. Therefore, these findings should be viewed as exploratory rather than conclusive.

## Conclusion

Overall, the main findings reported establish a significant association between elevated serum Cd levels and higher odds of migraine in a large Brazilian cohort, an association that may be influenced by smoking exposure. These findings underscore the need for greater awareness among clinicians and patients about smoking behavior. Future longitudinal studies with larger samples, however, are essential to confirm a causal relationship and to elucidate the sex-specific neurobiological mechanisms through which Cd may influence migraine pathogenesis.

## Data Availability

The ELSA-Brasil dataset is available upon request, based on a reasonable scientific justification and approval of a research proposal by the ELSA-Brasil’s Research Board.
